# 2-(4-Iodo­phen­yl)-1,2,3,4-tetra­hydro­isoquinoline-1-carbonitrile

**DOI:** 10.1107/S1600536811019830

**Published:** 2011-07-23

**Authors:** Yanni Ma, Lili Du, Qi Zhang, Fangjun Cao, Le Zhou

**Affiliations:** a College of Science, Northwest Agriculture and Forest University, Yangling 712100, People’s Republic of China; b College of Life Science, Northwest Agriculture and Forest University, Yangling 712100, People’s Republic of China

## Abstract

In the title compound, C_16_H_13_IN_2_, the benzene ring of the tetra­hydro­isoquinoline moiety makes a dihedral angle of 45.02 (9)° with the benzene ring of the 4-iodo­phenyl fragment. The N atom and the adjacent unsubstituted C atom of the tetra­hydro­isoquinoline unit are displaced by 0.294 (2) and 0.441 (3) Å, respectively, from the plane through the remaining eight C atoms. In the crystal, pairs of adjacent mol­ecules are linked into dimers by weak inter­molecular C—H⋯π inter­actions.

## Related literature

For the synthesis of the title compound, see: Ishii *et al.* (1985 ▶). For the biological activity of tetra­hydro­isoquinoline derivatives, see: Abe *et al.* (2005 ▶); Kamal *et al.* (2011 ▶); Lane *et al.* (2006 ▶); Liu *et al.* (2009 ▶); Storch *et al.* (2002 ▶); Wright *et al.* (1990 ▶).
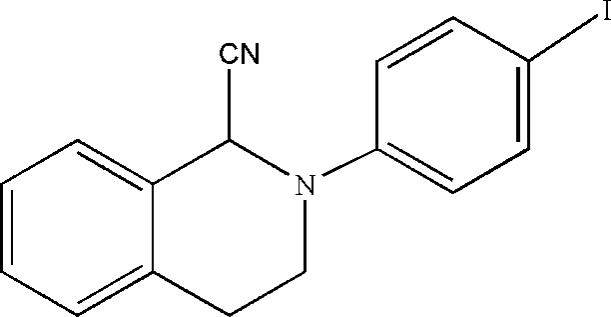


## Experimental

### 

#### Crystal data

C_16_H_13_IN_2_
*M* *_r_* = 360.18
Monoclinic, 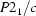

*a* = 7.347 (4) Å
*b* = 14.832 (8) Å
*c* = 13.149 (7) Å
β = 100.157 (6)°
*V* = 1410.5 (13) Å^3^
*Z* = 4
Mo *K*α radiation
μ = 2.26 mm^−1^
*T* = 296 K
0.32 × 0.17 × 0.15 mm

#### Data collection

Bruker APEXII CCD area-detector diffractometer
Absorption correction: multi-scan (*SADABS*; Sheldrick, 1996 ▶) *T*_min_ = 0.532, *T*_max_ = 0.728
10475 measured reflections
2604 independent reflections
2185 reflections with *I* > 2σ(*I*)
*R*_int_ = 0.018

#### Refinement

*R*[*F* ^2^ > 2σ(*F* ^2^)] = 0.024
*wR*(*F* ^2^) = 0.062
*S* = 1.00
2604 reflections
172 parameters
H-atom parameters constrained
Δρ_max_ = 0.55 e Å^−3^
Δρ_min_ = −0.42 e Å^−3^

### 

Data collection: *APEX2* (Bruker, 2004 ▶); cell refinement: *SAINT-Plus* (Bruker, 2004 ▶); data reduction: *SAINT-Plus*; program(s) used to solve structure: *SHELXS97* (Sheldrick, 2008 ▶); program(s) used to refine structure: *SHELXL97* (Sheldrick, 2008 ▶); molecular graphics: *SHELXTL* (Sheldrick, 2008 ▶); software used to prepare material for publication: *SHELXTL*.

## Supplementary Material

Crystal structure: contains datablock(s) global, I. DOI: 10.1107/S1600536811019830/rn2087sup1.cif

Structure factors: contains datablock(s) I. DOI: 10.1107/S1600536811019830/rn2087Isup2.hkl

Supplementary material file. DOI: 10.1107/S1600536811019830/rn2087Isup3.cml

Additional supplementary materials:  crystallographic information; 3D view; checkCIF report

## Figures and Tables

**Table 1:** Hydrogen-bond geometry (Å, °) *Cg* is the centroid of the C1–C6 ring.

*D*—H⋯*A*	*D*—H	H⋯*A*	*D*⋯*A*	*D*—H⋯*A*
C13—H13⋯*Cg*^i^	0.93	2.93	3.449 (4)	117

Symmetry code: (i) 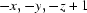.

**Fig. 1.**
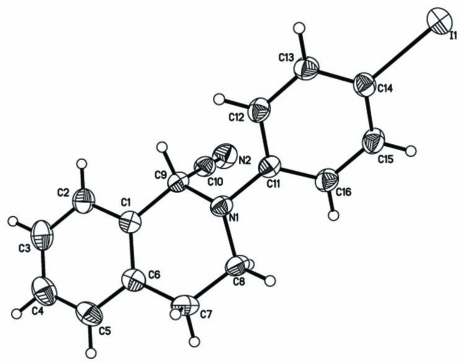
The molecular of the title compound showing 30% probability displacement ellipsoids for non-H atoms and the atom-numbering scheme.

**Fig. 2.**
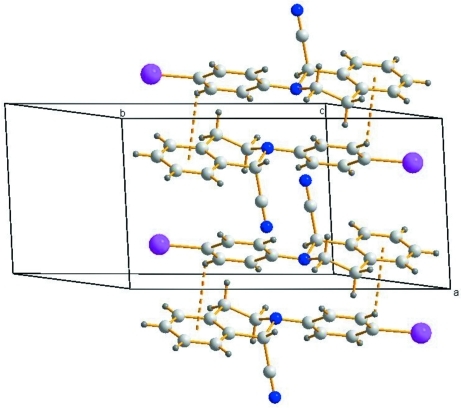
The dimer structure of the title compound showing C—H···π interactions.

## supplementary crystallographic information

### Comment

The tetrahydroisoquinoline derivatives have attracted great attention in recent years due to their neurotoxicity (Abe *et al.* 2005; Storch *et al.* 2002), antitumor activities (Lane *et al.* 2006; Wright *et al.* 1990), and antimicrobial activity (Kamal *et al.* 2011; Liu *et al.* 2009). We report here the synthesis and crystal structure of the title compound.

As shown in Fig. 1, benzene ring C1/C2/C3/C4/C5/C6 make a dihedral angle of 45.02 (9)° with benzene ring C11/C12/C13/C14/C15/C16. Atoms C7 and C9 are coplanar with benzene ring of the tetrahydroisoquinoline moiety. The conformation of the saturated six membered ring of the tetrahydroisoquinoline fragment is analyzed with respect to the plane formed by C1/C2/C3/C4/C5/C6/C7/C9, and the corresponding deviations are 0.441 (3) and 0.294 (2) Å for C8 and N1, respectively.

In the crystal structure, two adjacent molecules are linked into a dimer by weak intermolecular C—H···π interactions. The H···*Cg* distance is 2.930 Å with C···*Cg* of 3.449 (4) Å and C—H···*Cg* angle of 117°, as shown in Fig. 2. However, there are no weak C—H···I hydrogen bonds in the crystal structure of the title compound.

### Experimental

The title compound was synthesized according to the literature procedure (Ishii, *et al.* 1985), and crystals were obtained from a solution in ethyl acetate by slow evaporation at room temperature.

### Refinement

All of the non-hydrogen atoms were refined anisotropically. The hydrogen atoms were assigned with isotropic displacement factors *U*_iso_(H) = 1.2 times *U*_eq_(C), and included in the final refinement by using geometrical constraints, with C—H distances of 0.93 Å.

### Crystal data

**Table:**

C_16_H_13_IN_2_	*F*(000) = 704
*M**_r_* = 360.18	*D*_x_ = 1.696 Mg m^−^^3^
Monoclinic, *P*2_1_/*c*	Mo *K*α radiation, λ = 0.71073 Å
Hall symbol: -P 2ybc	Cell parameters from 4624 reflections
*a* = 7.347 (4) Å	θ = 2.8–26.3°
*b* = 14.832 (8) Å	µ = 2.26 mm^−^^1^
*c* = 13.149 (7) Å	*T* = 296 K
β = 100.157 (6)°	Block, colourless
*V* = 1410.5 (13) Å^3^	0.32 × 0.17 × 0.15 mm
*Z* = 4	

### Data collection

**Table:**

Bruker APEXII CCD area-detector diffractometer	2604 independent reflections
Radiation source: fine-focus sealed tube	2185 reflections with *I* > 2σ(*I*)
graphite	*R*_int_ = 0.018
φ and ω scans	θ_max_ = 25.5°, θ_min_ = 2.8°
Absorption correction: multi-scan (*SADABS*; Sheldrick, 1996)	*h* = −8→8
*T*_min_ = 0.532, *T*_max_ = 0.728	*k* = −17→17
10475 measured reflections	*l* = −15→15

### Refinement

**Table:**

Refinement on *F*^2^	Primary atom site location: structure-invariant direct methods
Least-squares matrix: full	Secondary atom site location: difference Fourier map
*R*[*F*^2^ > 2σ(*F*^2^)] = 0.024	Hydrogen site location: inferred from neighbouring sites
*wR*(*F*^2^) = 0.062	H-atom parameters constrained
*S* = 1.00	*w* = 1/[σ^2^(*F*_o_^2^) + (0.0295*P*)^2^ + 0.8261*P*] where *P* = (*F*_o_^2^ + 2*F*_c_^2^)/3
2604 reflections	(Δ/σ)_max_ < 0.001
172 parameters	Δρ_max_ = 0.55 e Å^−^^3^
0 restraints	Δρ_min_ = −0.42 e Å^−^^3^

### Special details

**Table:**

Geometry. All e.s.d.'s (except the e.s.d. in the dihedral angle between two l.s. planes)are estimated using the full covariance matrix. The cell e.s.d.'s are takeninto account individually in the estimation of e.s.d.'s in distances, anglesand torsion angles; correlations between e.s.d.'s in cell parameters are onlyused when they are defined by crystal symmetry. An approximate (isotropic)treatment of cell e.s.d.'s is used for estimating e.s.d.'s involving l.s. planes.
Refinement. Refinement of *F*^2^ against ALL reflections. The weighted *R*-factor *wR* and goodness of fit *S* are based on *F*^2^, conventional *R*-factors *R* are based on *F*, with *F* set to zero for negative *F*^2^. The threshold expression of *F*^2^ > σ(*F*^2^) is used only for calculating *R*-factors(gt) *etc*. and is not relevant to the choice of reflections for refinement. *R*-factors based on *F*^2^ are statistically about twice as large as those based on *F*, and *R*- factors based on ALL data will be even larger.

### Fractional atomic coordinates and isotropic or equivalent isotropic displacement parameters (Å^2^)

**Table:**

	*x*	*y*	*z*	*U*_iso_*/*U*_eq_	
C1	0.2714 (3)	−0.15209 (17)	0.6261 (2)	0.0437 (6)	
C2	0.3306 (4)	−0.21738 (19)	0.5630 (2)	0.0546 (7)	
H2	0.3822	−0.2001	0.5064	0.065*	
C3	0.3126 (4)	−0.3078 (2)	0.5845 (3)	0.0669 (8)	
H3	0.3516	−0.3511	0.5420	0.080*	
C4	0.2371 (4)	−0.3340 (2)	0.6686 (3)	0.0689 (9)	
H4	0.2256	−0.3949	0.6832	0.083*	
C5	0.1793 (4)	−0.2698 (2)	0.7306 (2)	0.0600 (8)	
H5	0.1289	−0.2879	0.7874	0.072*	
C6	0.1940 (4)	−0.17800 (19)	0.7107 (2)	0.0478 (6)	
C7	0.1195 (4)	−0.1080 (2)	0.7750 (2)	0.0573 (7)	
H7A	−0.0136	−0.1043	0.7538	0.069*	
H7B	0.1448	−0.1265	0.8468	0.069*	
C8	0.2025 (4)	−0.01582 (19)	0.76536 (19)	0.0488 (6)	
H8A	0.1375	0.0287	0.7995	0.059*	
H8B	0.3314	−0.0158	0.7985	0.059*	
C9	0.2960 (3)	−0.05317 (17)	0.60181 (19)	0.0413 (6)	
H9	0.2557	−0.0446	0.5273	0.050*	
C10	0.4980 (4)	−0.02988 (18)	0.6277 (2)	0.0467 (6)	
C11	0.2096 (3)	0.10006 (16)	0.63235 (19)	0.0393 (5)	
C12	0.1592 (4)	0.12960 (18)	0.53047 (19)	0.0448 (6)	
H12	0.1136	0.0883	0.4790	0.054*	
C13	0.1760 (4)	0.21893 (18)	0.5050 (2)	0.0485 (6)	
H13	0.1421	0.2377	0.4368	0.058*	
C14	0.2433 (3)	0.28069 (17)	0.5812 (2)	0.0446 (6)	
C15	0.2939 (4)	0.2529 (2)	0.6825 (2)	0.0523 (6)	
H15	0.3388	0.2945	0.7337	0.063*	
C16	0.2777 (4)	0.16344 (18)	0.7076 (2)	0.0495 (6)	
H16	0.3128	0.1451	0.7759	0.059*	
I1	0.26329 (3)	0.417129 (13)	0.542901 (16)	0.06623 (10)	
N1	0.1875 (3)	0.00726 (14)	0.65543 (15)	0.0412 (5)	
N2	0.6490 (4)	−0.01027 (19)	0.6491 (2)	0.0653 (7)	

### Atomic displacement parameters (Å^2^)

**Table:**

	*U*^11^	*U*^22^	*U*^33^	*U*^12^	*U*^13^	*U*^23^
C1	0.0390 (13)	0.0450 (14)	0.0460 (14)	0.0045 (11)	0.0048 (11)	0.0015 (11)
C2	0.0525 (16)	0.0515 (16)	0.0595 (17)	0.0038 (13)	0.0094 (13)	−0.0053 (13)
C3	0.0638 (19)	0.0478 (17)	0.087 (2)	0.0097 (15)	0.0064 (17)	−0.0124 (16)
C4	0.065 (2)	0.0480 (18)	0.088 (2)	0.0024 (15)	−0.0024 (18)	0.0113 (17)
C5	0.0554 (17)	0.0551 (18)	0.0669 (19)	−0.0026 (14)	0.0036 (14)	0.0177 (15)
C6	0.0408 (14)	0.0522 (15)	0.0484 (15)	0.0000 (12)	0.0024 (11)	0.0063 (12)
C7	0.0628 (18)	0.0627 (18)	0.0506 (16)	0.0028 (14)	0.0217 (14)	0.0126 (13)
C8	0.0569 (16)	0.0543 (16)	0.0377 (13)	0.0071 (13)	0.0150 (12)	0.0013 (12)
C9	0.0431 (14)	0.0449 (13)	0.0363 (13)	0.0030 (11)	0.0085 (10)	−0.0005 (10)
C10	0.0496 (17)	0.0475 (15)	0.0465 (15)	0.0045 (12)	0.0182 (12)	0.0000 (12)
C11	0.0343 (13)	0.0451 (14)	0.0391 (13)	0.0060 (10)	0.0078 (10)	−0.0017 (10)
C12	0.0452 (15)	0.0470 (15)	0.0392 (13)	0.0040 (12)	−0.0012 (11)	−0.0036 (11)
C13	0.0531 (16)	0.0501 (15)	0.0397 (13)	0.0061 (12)	0.0015 (12)	0.0038 (12)
C14	0.0436 (14)	0.0405 (13)	0.0501 (15)	0.0037 (11)	0.0095 (11)	0.0003 (11)
C15	0.0573 (16)	0.0515 (16)	0.0456 (14)	−0.0012 (13)	0.0023 (12)	−0.0090 (12)
C16	0.0570 (16)	0.0519 (16)	0.0364 (13)	0.0039 (13)	−0.0004 (12)	0.0000 (12)
I1	0.09151 (18)	0.04555 (13)	0.06353 (15)	−0.00255 (10)	0.01885 (11)	0.00301 (9)
N1	0.0436 (11)	0.0441 (12)	0.0374 (11)	0.0057 (9)	0.0114 (9)	0.0007 (9)
N2	0.0508 (16)	0.0792 (19)	0.0693 (17)	−0.0032 (14)	0.0199 (13)	−0.0087 (14)

### Geometric parameters (Å, °)

**Table:**

C1—C6	1.391 (4)	C8—H8B	0.9700
C1—C2	1.393 (4)	C9—N1	1.461 (3)
C1—C9	1.519 (4)	C9—C10	1.503 (4)
C2—C3	1.381 (4)	C9—H9	0.9800
C2—H2	0.9300	C10—N2	1.133 (3)
C3—C4	1.378 (5)	C11—C16	1.392 (4)
C3—H3	0.9300	C11—C12	1.396 (3)
C4—C5	1.368 (5)	C11—N1	1.425 (3)
C4—H4	0.9300	C12—C13	1.378 (4)
C5—C6	1.395 (4)	C12—H12	0.9300
C5—H5	0.9300	C13—C14	1.383 (4)
C6—C7	1.501 (4)	C13—H13	0.9300
C7—C8	1.511 (4)	C14—C15	1.381 (4)
C7—H7A	0.9700	C14—I1	2.097 (3)
C7—H7B	0.9700	C15—C16	1.378 (4)
C8—N1	1.471 (3)	C15—H15	0.9300
C8—H8A	0.9700	C16—H16	0.9300
			
C6—C1—C2	119.9 (3)	H8A—C8—H8B	108.3
C6—C1—C9	121.0 (2)	N1—C9—C10	110.6 (2)
C2—C1—C9	119.0 (2)	N1—C9—C1	113.3 (2)
C3—C2—C1	120.1 (3)	C10—C9—C1	108.9 (2)
C3—C2—H2	119.9	N1—C9—H9	108.0
C1—C2—H2	119.9	C10—C9—H9	108.0
C2—C3—C4	120.3 (3)	C1—C9—H9	108.0
C2—C3—H3	119.8	N2—C10—C9	177.9 (3)
C4—C3—H3	119.8	C16—C11—C12	118.1 (2)
C5—C4—C3	119.5 (3)	C16—C11—N1	122.8 (2)
C5—C4—H4	120.2	C12—C11—N1	119.1 (2)
C3—C4—H4	120.2	C13—C12—C11	121.0 (2)
C4—C5—C6	121.7 (3)	C13—C12—H12	119.5
C4—C5—H5	119.2	C11—C12—H12	119.5
C6—C5—H5	119.2	C12—C13—C14	119.8 (2)
C1—C6—C5	118.4 (3)	C12—C13—H13	120.1
C1—C6—C7	120.0 (2)	C14—C13—H13	120.1
C5—C6—C7	121.5 (3)	C13—C14—C15	120.1 (2)
C8—C7—C6	112.7 (2)	C13—C14—I1	119.8 (2)
C8—C7—H7A	109.1	C15—C14—I1	120.1 (2)
C6—C7—H7A	109.0	C16—C15—C14	119.9 (2)
C8—C7—H7B	109.0	C16—C15—H15	120.0
C6—C7—H7B	109.1	C14—C15—H15	120.0
H7A—C7—H7B	107.8	C15—C16—C11	121.0 (2)
N1—C8—C7	109.4 (2)	C15—C16—H16	119.5
N1—C8—H8A	109.8	C11—C16—H16	119.5
C7—C8—H8A	109.8	C11—N1—C9	113.42 (19)
N1—C8—H8B	109.8	C11—N1—C8	116.3 (2)
C7—C8—H8B	109.8	C9—N1—C8	112.3 (2)
			
C6—C1—C2—C3	−0.1 (4)	N1—C11—C12—C13	179.1 (2)
C9—C1—C2—C3	178.9 (3)	C11—C12—C13—C14	−0.1 (4)
C1—C2—C3—C4	−0.4 (5)	C12—C13—C14—C15	0.1 (4)
C2—C3—C4—C5	0.3 (5)	C12—C13—C14—I1	−178.6 (2)
C3—C4—C5—C6	0.2 (5)	C13—C14—C15—C16	0.2 (4)
C2—C1—C6—C5	0.5 (4)	I1—C14—C15—C16	178.9 (2)
C9—C1—C6—C5	−178.4 (2)	C14—C15—C16—C11	−0.5 (4)
C2—C1—C6—C7	−176.2 (3)	C12—C11—C16—C15	0.4 (4)
C9—C1—C6—C7	4.8 (4)	N1—C11—C16—C15	−178.8 (2)
C4—C5—C6—C1	−0.6 (4)	C16—C11—N1—C9	−119.8 (3)
C4—C5—C6—C7	176.1 (3)	C12—C11—N1—C9	61.0 (3)
C1—C6—C7—C8	−22.5 (4)	C16—C11—N1—C8	12.6 (3)
C5—C6—C7—C8	160.9 (3)	C12—C11—N1—C8	−166.6 (2)
C6—C7—C8—N1	50.9 (3)	C10—C9—N1—C11	57.7 (3)
C6—C1—C9—N1	−16.1 (3)	C1—C9—N1—C11	−179.7 (2)
C2—C1—C9—N1	165.0 (2)	C10—C9—N1—C8	−76.6 (3)
C6—C1—C9—C10	107.4 (3)	C1—C9—N1—C8	46.0 (3)
C2—C1—C9—C10	−71.5 (3)	C7—C8—N1—C11	162.7 (2)
C16—C11—C12—C13	−0.1 (4)	C7—C8—N1—C9	−64.4 (3)

### Hydrogen-bond geometry (Å, °)

**Table:**

Cg is the centroid of the C1–C6 ring.

**Table:**

*D*—H···*A*	*D*—H	H···*A*	*D*···*A*	*D*—H···*A*
C13—H13···Cg^i^	0.93	2.93	3.449 (4)	117

Symmetry codes: (i) −*x*, −*y*, −*z*+1.
